# Pollination by long‐proboscid horseflies and its implications for reproductive isolation among coflowering *Satyrium* orchids in South Africa

**DOI:** 10.1002/ajb2.70221

**Published:** 2026-06-12

**Authors:** Steven D. Johnson, Matthew Moir, Ethan Newman, Timotheüs Van der Niet

**Affiliations:** ^1^ Centre for Functional Biodiversity, School of Life Sciences University of KwaZulu‐Natal Pietermaritzburg Private Bag X01 Scottsville 3209 South Africa; ^2^ Department of Botany Rhodes University PO Box 94 Makhanda 6140 South Africa; ^3^ Department of Biological Sciences University of Cape Town Private Bag X3 Rondebosch 7701 South Africa; ^4^ Present address: Department of Botany and Zoology Stellenbosch University Matieland 7602 South Africa

**Keywords:** ecotypes, flower colour, fly pollination, Grant‐Stebbins model, hawkmoths, hybridization, long‐proboscid flies, Orchidaceae, Philoliche, Tabanidae

## Abstract

**Premise:**

Floral adaptations to pollinators can drive lineage diversification and promote coexistence of species. We investigated the reproductive biology of *Satyrium longicolle*, a South African orchid that we hypothesized to belong to a long‐proboscid horsefly pollination guild and examined overlap of pollinators and floral traits among coflowering sympatric *Satyrium* species.

**Methods:**

We observed pollinators across different sites and years, carried out experimental pollinations, and measured spectral reflectance, scent chemistry, floral dimensions and nectar properties for both *S. longicolle*, and sympatric congeners and other species belonging to the horsefly pollination guild. Spectral reflectance was analyzed in appropriate insect vision models.

**Results:**

*Satyrium longicolle* has weakly scented flowers that are pollinated diurnally by long‐proboscid *Philoliche* horseflies, which ignored the strongly scented flowers of sympatric moth‐pollinated congeners. The cream UV‐reflecting flowers of S. *longicolle* and other guild members differed in color, but not achromatic contrast, from the white UV‐absorbing flowers of hawkmoth‐pollinated species. Although spur lengths and nectar properties are similar among the sympatric *Satyrium* species, spurs of *S. longicolle* are horizontal, while spurs of hawkmoth‐pollinated congeners are pendant, coinciding with different foraging behaviors of the respective pollinators. Spur length of *S. longicolle* co‐varied geographically with horsefly proboscis length. The orchid is self‐compatible but reliant on pollinators for seed production. Crosses with a sympatric moth‐pollinated congener resulted in seeds with embryos.

**Conclusions:**

The novel evolution of a specialized horsefly pollination system within *Satyrium* contributes to phenotypic diversity in the genus and results in strong ethological reproductive isolation with sympatric moth‐pollinated congeners.

Floral adaptation to pollinators is recognized as a key driver of diversification in the angiosperms (Grant, 1949; Stebbins, 1970). This mode of diversification is evident from convergent floral evolution among unrelated plants that share pollinator groups (Fenster et al., 2004), correlated transitions in floral traits and pollinators reconstructed using phylogenies (Van der Niet and Johnson, 2012), and experimental studies of floral function and phenotypic selection (Harder and Johnson, 2009; Moir et al., 2025). All of these approaches require natural history information on the pollinators and floral traits of plants (Van der Niet, 2021). Therefore, the discovery and documentation of new pollination systems in plants can lead to important advances in our knowledge of pollinator‐driven diversification.

The flora of South Africa is renowned for its diversity of pollination systems, and many of these are found only in this region or play a disproportionate role in this region (Johnson, 2010). One such system involves the role of long‐proboscid flies as pollinators. These flies in the families Tabanidae and Nemestrinidae are responsible for the pollination of several hundred plant species in the region and one of the most outstanding features of these systems is the high degree of specialization (Goldblatt and Manning, 2000). In many cases, plants are pollinated by a single long‐proboscid fly species or just two or three functionally similar species (Johnson and Steiner, 2003; Johnson, 2010). Specialization seems to be a general feature of long‐proboscid fly pollination systems, even where they occur outside of southern Africa, such as the plant species in the Himalayas that is pollinated by a long‐proboscid horsefly species (Paudel et al., 2015, 2016). Various groups of long‐proboscid flies represent unique pollinator niches that have been key drivers of diversification in the South African flora (Johnson, 2010). Plants that are distributed beyond the range of a particular fly species often show regional floral specialization to different fly species or other functional pollinator groups outside the range (Johnson and Steiner, 1997; Pauw et al., 2009; Newman et al., 2015). This process of ecotype formation is a key step that can lead to pollinator‐driven speciation (Johnson, 2025a).

Apart from their role in driving phenotypic diversification, specialized pollination niches can also play an important role in mediating plant species coexistence. Adaptation to these niches can promote reproductive isolation among congeneric species if they have non‐overlapping sets of pollinators (McCarren et al., 2023; Newman and Johnson, 2025). For instance, differences in floral scent and the angle of floral tubes can contribute to reproductive isolation among species pollinated by long‐proboscid flies and closely related congeners pollinated by hawkmoths (Campbell et al., 2016).

Traits that are characteristic of plants pollinated by long‐proboscid flies include long floral tubes; minimal emission of floral scent; cream, pink, or purple flower colors; and conspicuous nectar guides (Goldblatt and Manning, 2000; Campbell et al., 2016; McCarren et al., 2023; Johnson et al., 2025). The orientation of flowers often differs between flowers pollinated by tabanid flies that have their proboscis fixed in a horizontal position, and nemestrinid flies that are capable of swiveling their proboscis downward and can feed on flowers with vertical floral tubes (McCarren et al., 2022, 2023). Convergent evolution of flower color has been noted in several guilds of plants pollinated by long‐proboscid flies, and a reflectance spectrum that includes a significant UV component is a general feature of flowers pollinated by these flies (McCarren et al., 2021). The functional role of nectar guides has been demonstrated experimentally in a study of a *Lapeirousia* (Iridaceae) species pollinated by long‐proboscid flies (Hansen et al., 2012).

Pollination by long‐proboscid flies has been documented in several orchid species in South Africa. Several species in the genus *Disa* have been shown to be pollinated exclusively by tabanid flies (Johnson and Steiner, 1997; Johnson, 2000; Johnson and Morita, 2006). All these cases involve floral mimicry by deceptive orchids that phenotypically resemble rewarding non‐orchid food plants. However, although pollination systems are known for 38 *Satyrium* taxa, specialization for tabanid fly pollination has not previously been recorded in the genus in which almost all species are nectar rewarding. The present study was initially motivated by a citizen scientist photograph (reproduced by Liltved and Johnson, 2012) of a resting horsefly (*Philoliche gulosa*) carrying pollinaria that matched those of *Satyrium longicolle* Lindl., which was flowering in the near vicinity in the Langkloof Valley. On the basis of this photograph and the similarity in some of the floral traits of this orchid (e.g., its long horizontal spurs and nectar guide marks, which are very unusual in *Satyrium*) to those of other plants pollinated by horseflies (Tabanidae), we hypothesized that *S. longicolle* may be specialized for pollination by tabanid flies. *Satyrium longicolle* often occurs in sympatry with other *Satyrium* species, such as *S. acuminatum* Lindl. and *S. membranaceum* Sw. (Figure 1), that have been shown to be pollinated by moths (Van der Niet et al., 2015; Botes et al., 2020). Some *Satyrium* species are interfertile, and several natural hybrids have been documented (Ellis and Johnson, 1999; Johnson, 2018). Differences in pollination systems among *Satyrium* species are important for promoting their sustained coexistence in sympatry (Ellis and Johnson, 1999), and we therefore hypothesized that pollinators of *S. longicolle* and its congeners would show high levels of foraging fidelity in mixed stands, resulting in strong pollinator‐mediated reproductive isolation.

**Figure 1:**
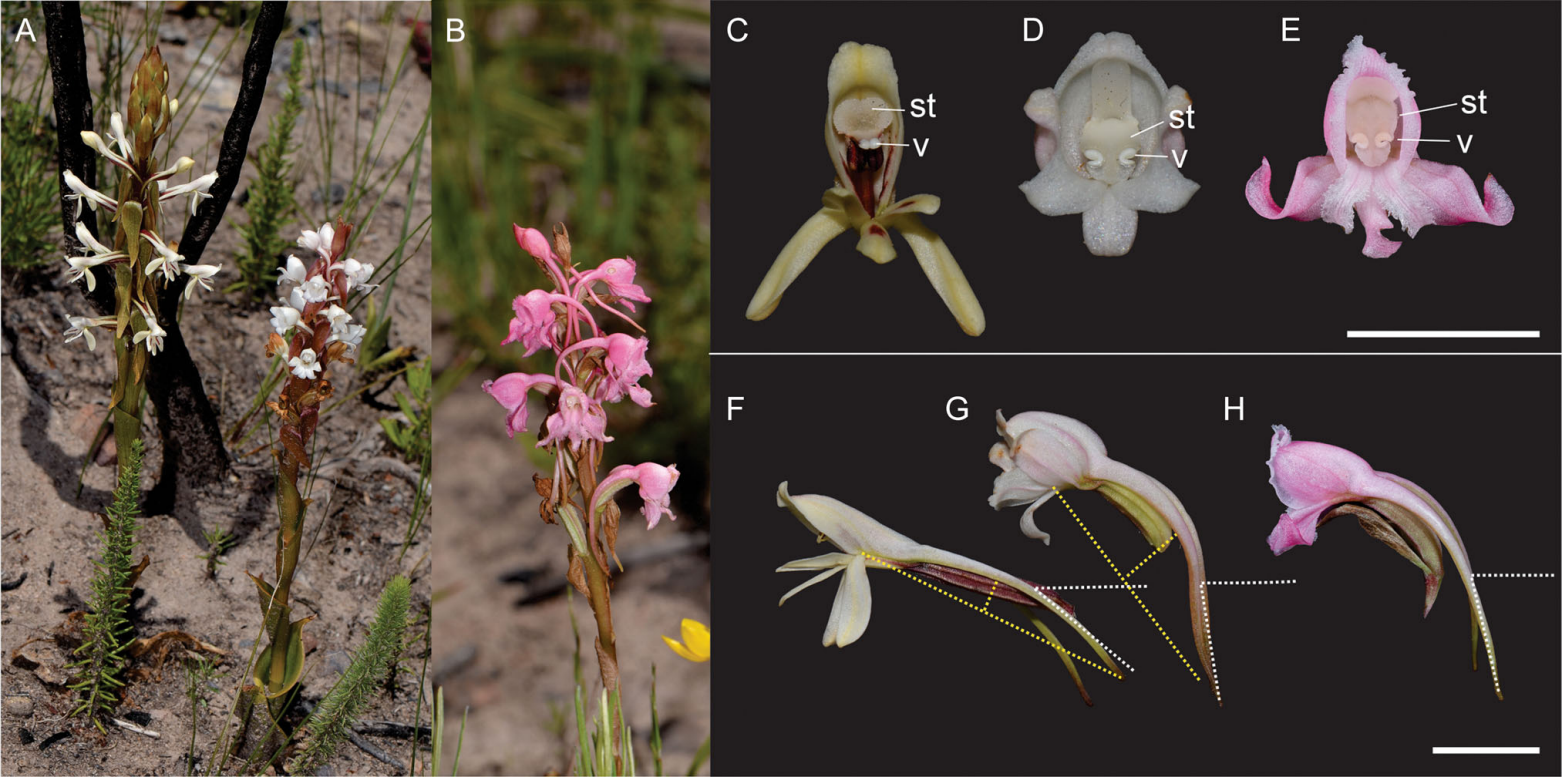
Morphology of three *Satyrium* species that coflower in sympatry. (A) *S. longicolle* (left) and *S. acuminatum* (right) flowering side‐by‐side. (B) *S. membranaceum* coflowering in the same community. (C) *S. longicolle* front view. (D) *S. acuminatum* front view. (E) *S. membranaceum* front view. (F) *S. longicolle* side view. (G) *S. acuminatum* side view. (H) *S. membranaceum* side view. st = stigma, v = viscidia. Scale: 10 mm. Dotted lines indicate measurements of spur angles (see the section on Morphology, nectar, and flowering phenology in Materials and Methods for details).

To test these hypotheses, we addressed the following questions: (1) Is *S. longicolle* pollinated by tabanid horseflies across its distribution range? (2) Do tabanid flies transfer pollen among flowers efficiently and is pollen flow exclusively diurnal? (3) Are floral traits, such as morphology, color and scent, consistent with the general floral syndrome of specialized pollination by tabanid flies? (4) Do regional differences in floral traits of the orchid correlate with differences in pollinator assemblages? (5) Do tabanid flies show fidelity to this orchid species when foraging in mixed stands of congeners? (6) Are plants of *S. longicolle* dependent on pollinators for seed production, and are they interfertile with congeners?

## MATERIALS AND METHODS

### Study species

**Figure 2:**
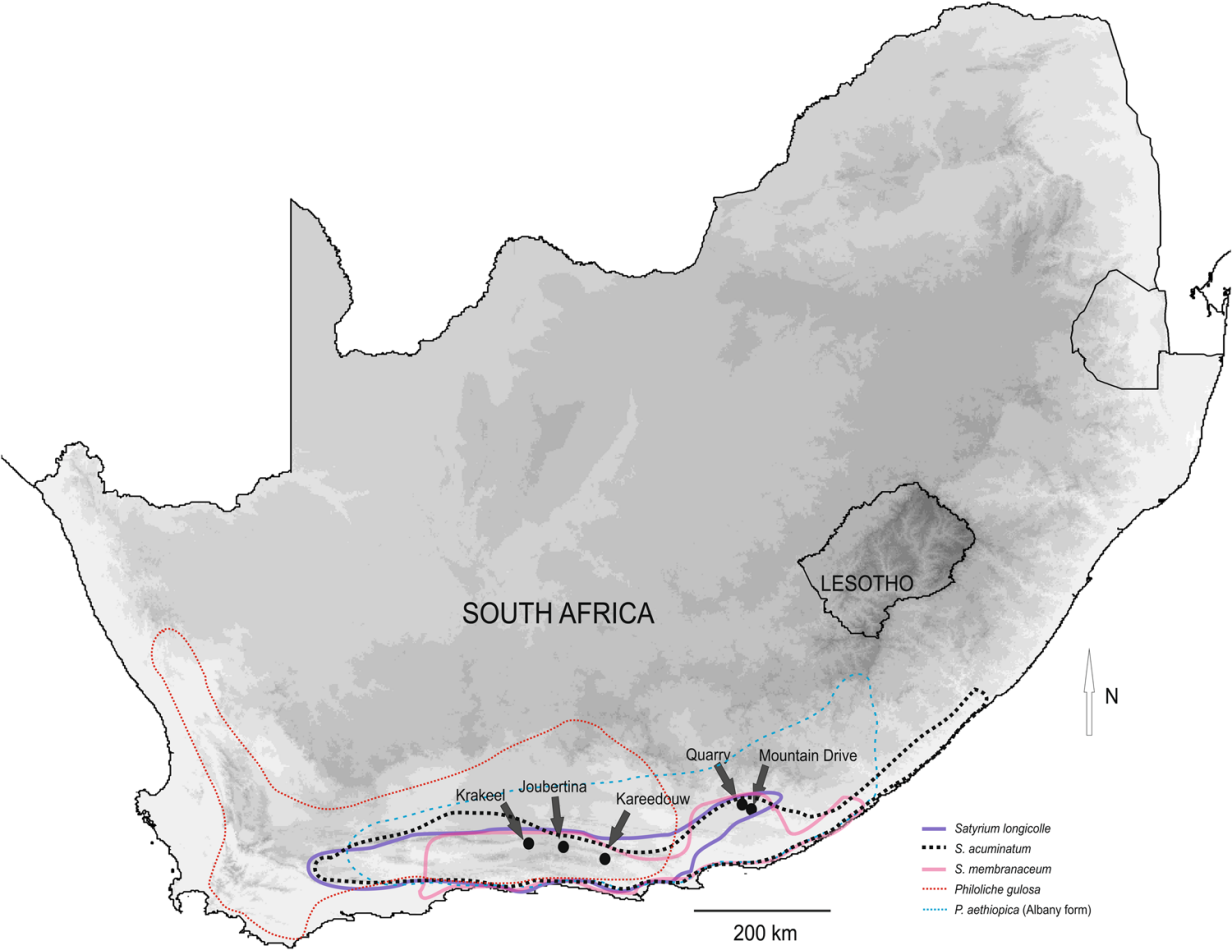
Study sites and distributions of three *Satyrium* species and the two *Philoliche* horsefly species that pollinate *S. longicolle*. *S. acuminatum* and *S. membranaceum* are pollinated by moths that occur widely across the region.

*Satyrium longicolle* Lindl. (Orchidaceae) occurs in the southern Cape region of South Africa, where it generally grows in renosterveld and fynbos habitats of the coastal forelands to the first range of mountains that run parallel to the coast at elevations up to 750 m (Figure 2). At its eastern margin, it occurs in grassy habitats. The distribution range is characterized by all year rainfall in the west to summer rainfall in the east. Flowering of *S. longicolle* occurs during the austral late spring and summer when its leaves are withered (Figure 1A), and flowering is stimulated by fire. The flowers of *S. longicolle* are notable for nectar guide patterns, including purple lines on the perianth and purple anther sacs (Figure 1C) and for the relatively straight and horizontal spurs (Figure 1F). The flowers have globose viscidia, which in *Satyrium* are often associated with placement of pollinaria on the body of insects as opposed to the proboscis (Johnson, 1997a). The closest relatives of *S. longicolle* differ markedly in floral traits (Van der Niet and Linder, 2008). Known pollination systems of the closest relatives include bee pollination in the short‐spurred *S. erectum* Sw. (Johnson, 1996) and moth pollination in the long‐spurred and fragrant *S. pallens* S.D. Johnson & Kurzweil, *S. bicorne* (L.) Thunb., and *S. situsanguinum* Van der Niet & Liltved (Van der Niet et al., 2015).

*Satyrium longicolle* has often been recorded to coflower in sympatry with *Satyrium acuminatum* and *S. membranaceum* (Liltved and Johnson, 2012), and these three congeners share habitats and distributions (Figure 2). The last two species have scented flowers and are pollinated at night by moths (Van der Niet et al., 2015; Botes et al., 2020). They have a large rostellum with notched lateral viscidia (Figure 1D,E). Notched viscidia in *Satyrium* function to clasp around the proboscis of long‐tongued insects such as moths (Johnson, 1997a).

### Study sites

Fieldwork was carried out in 2012, 2016, 2017, and 2022 at five sites in the Eastern Cape Province of South Africa. Three of these sites—Krakeel (–33.8415, 23.73250), Joubertina (–33.81914, 23.865079), and Kareedouw (–33.940986, 24.335702)—are in the Langkloof region of the southern Cape region (Figure 2). Due to the fire‐stimulated flowering of all three *Satyrium* species, fieldwork took place opportunistically after fires in the renosterveld and fynbos vegetation at these sites. Two of the sites—Quarry (–33.251156, 26.578503) and Mountain Drive (–33.328602, 26.575023)—are situated close to the town of Makhanda (formerly Grahamstown) at the easternmost margin of the distribution of *S. longicolle* (Figure 2). Pollinator observations, measurements of pollination success, and plant measurements were carried out at all five sites. *Satyrium longicolle* coflowered with *S. acuminatum* and *S. membranaceum* at all of the study sites, apart from Makhanda where *S. acuminatum* was absent. The largest populations were found at the Kareedouw site where ca 600 flowering *S. longicolle* plants were interspersed with ca 200 coflowering plants of *S. acuminatum* and ca 80 coflowering plants of *S. membranaceum* in an area of ca 200 m by 150 m. Though flowering overlaps for all three species, flowering of *S. membranaceum* occurs slightly earlier than that of *S. acuminatum* and *S. longicolle* (Appendix S1). Some data could therefore only be collected for the last two species at this study site. Voucher specimens from the study sites are deposited in the Bews herbarium (NU) in Pietermaritzburg.

### Pollinator observations

Pollinators in the Langkloof region were observed at Krakeel from 25–26 October 2016, at Joubertina from 9–11 and 13 November 2017 and at Kareedouw from 18–22 November 2017 and on 12 December 2017 and in the Makhanda region on 21 November 2022 at the Quarry site and 23–24, 29–30 November, and 1 and 7–9 December 2022 at the Mountain Drive site. In general, observations were carried out from 08:00 to 14:00 on warm, clear days. At Krakeel and Kareedouw, we also carried out observations in the evenings from c. 18:00 until 21:00. We used flashlights with red filters to enable direct observations of moth behavior. Pollinators were observed over a total of 22 days across the sites.

We recorded the details of pollinator foraging bouts, including the *Satyrium* species visited, number of flowers probed per plant, whether one or both of the floral spurs were probed, the total time of the visit and whether pollinaria were present on insects. Due to the proximity of the entrance to the two spurs (only separated by the narrow column foot), it was not possible to assess which of the two spurs was probed. Although the focus was on *S. longicolle*, we also observed visitors to flowers of *S. acuminatum* and *S. membranaceum* at the Kareedouw site. We took close‐up photographs of flower visitors, which allowed us to establish the number and position of pollinaria on their bodies and, in the case of horseflies, to sex the individuals (using their eye spacing). To enable recording of flower visitors over 24 h, that included times when we were not present in the study population, we used four motion‐activated cameras (Nature View 119740, Bushnell, Overland Park, KS, USA) at the Kareedouw site in 2017. These cameras can detect long‐proboscid flies and moths when fitted with close‐up lenses that allow the cameras to be placed 20–45 cm from flowers (Johnson et al., 2019). Finally, representative insect visitors were captured and deposited in the collections at the University of KwaZulu‐Natal and the Albany Museum in Makhanda. The body length and proboscis length of these insects were measured with digital calipers. To calculate the effective (extended) functional proboscis length, we measured the distance from the clypeus to the tip of the proboscis and added 7 mm for *P. gulosa* and 7.3 mm for *P. aethiopica*, which is the known length of the prementum (sclerotized part of the labrum) of these fly species, as calculated by Morita (2011). During feeding, these flies can extend their proboscis through elongation of the rostral ventral membrane up to the length of the prementum (Morita, 2011). To assess the flight periods of *P. gulosa* and *P. aethiopica*, we used collection dates for specimens of these flies in the Natal Museum and records from iNaturalist (https://www.inaturalist.org/taxa/683310-Philoliche-gulosa, https://www.inaturalist.org/taxa/631923-Philoliche-aethiopica).

### Floral traits

#### Morphology, nectar, and flowering phenology

To assess the functional fit between flowers and pollinators in flowers of *S. longicolle* and its sympatric congeners, we measured floral spur length of 21–94 (median = 30) plants per population. We used digital calipers to measure the distance from the stigma to the tip of the spur as a measure of flower depth. As a measure of the curvature of the spur, we photographed 3–11 inflorescences (median = 10) of each species at the Kareedouw site with a ruler for scale and used ImageJ 1.54 g (National Institutes of Health, Bethesda, MD, USA) to measure the chord (straight distance between the entrance and tip of the spur) and the maximum deviation of a line tangential from the chord to the spur (Figure 1F–H). The ratio of the chord to the length of the deviation from the chord was used as an estimate of curvature. As a measure of spur recurvature, we measured the angle of the distal half of the spur relative to the horizontal plane (Figure 1F–H). Other floral traits measured for the three species included pollinarium length, pollinarium caudicle length, and the number of per pollinarium. For massulae, pollinaria were dabbed onto clear sticky tape and the masulae on the tape were counted using a 10× hand lens. Floral dimensions were compared among all populations of *S. longicolle* and among the three species in the Langkloof valley using Gaussian generalized linear models (GLMs). Significance was assessed using likelihood ratios and we used the Dunn–Šidák procedure (Kirk, 1995) for post hoc comparisons of mean values. Unless otherwise stated, statistical procedures were implemented in SPSS 29 (IBM Corp., Armonk, NY, USA).

To estimate availability of nectar rewards in flowers of the study species, we measured the volume of the standing crop of nectar in one randomly chosen spur in each of 122 flowers of *S. longicolle*, eight flowers of *S. acuminatum* and eight flowers of *S. membranaceum* using 5‐µL pipettes. The sugar concentration was determined with a 0–50% hand‐held refractometer (Bellingham & Stanley model 45‐81, Xylem Analytics, Weilheim, Germany).

To characterize the flowering phenology, we used the citizen science platform iNaturalist (www.inaturalist.org). Only observations that were annotated to include flowers were used, to avoid including observations of nonflowering individuals in the assessment of phenology. Records were graphed using a frequency distribution.

#### Spectral reflectance

To assess the visual signals that may be used by pollinators, we measured the spectral reflectance of the labellum, lateral sepals, dorsal sepal, and guide markings (if any) for *S. longicolle*, *S. acuminatum*, *S. membranaceum*, and representatives of other flowering plants with which these species shared flower visitors. Spectral measurements were obtained using an Ocean Optics S2000 spectrometer (Ocean Optics, Dunedin, FL, USA) and the instrument settings described by Johnson and Andersson (2002).

To visualize the spectral profiles of floral parts, we generated aggregated spectral reflectance curves using the function aggplot. To assess the perception of floral color in pollinator vision, we used visual modelling with the functions vismodel and colspace and D65 daylight illuminance and a green background spectrum derived from a general sample of Eastern Cape foliage. For plotting, spectral loci were first modelled in fly vision using the color opponent coding vision model (Troje, 1993) as modified by Garcia et al. (2022) to incorporate photoreceptor sensitivities of *Eristalis tenax* (Syrphidae), which is more closely related to *Philoliche* than is *Musca domestica* (Muscidae), which was used in the original Troje model. To assess the perception of floral color in hawkmoth vision, we modeled spectral loci in trichromatic color space based on the photoreceptor sensitivities of the nocturnal hawkmoth *Deilephila elpenor* (Höglund et al., 1973; Johnson et al., 2014). Because there is evidence that certain pollinators, particularly hawkmoths, can use achromatic contrast information to make flower choices (Kelber, 2005; Van Der Kooi and Kelber 2022), we calculated achromatic contrast values for the green receptor of *D. elpenor* and *E. tenax*. For *E. tenax*, we used a tetrahedral color space model, rather than the Troje model, which allows for more direct comparison with trichromatic color space. In all visual models, receptor quantum catches were transformed using a Naka–Rushton function (Naka and Rushton, 1966).

To assess whether the distribution of spectral loci modeled in Troje color space potentially reflects fly‐perceptible color differences of flowers within and between fly and moth pollination guilds, we computed pairwise chromatic contrast values (dS) using the function coldist and compared them against the “functional discrimination” threshold for *E. tenax* (0.021 Troje units) (Hannah et al., 2019; Garcia et al., 2022). To compare achromatic contrast values for putatively fly‐pollinated *S. longicolle* and the hawkmoth‐pollinated species *S. acuminatum*, and *S. membranaceum*, and representatives of other flowering plants with which these species shared flower visitors, we used the function coldist to extract achromatic contrast values (dL) of spectral loci against the background spectrum in trichromatic hawkmoth visual space and tetrachromatic fly visual space. These color distances were analyzed using a Gaussian GLMM with pollinator type as a fixed factor and species (or ecotype) as a random factor to account for non‐independence of samples of the same species. All spectral reflectance and visual modelling analyses were conducted using the R package pavo (Maia et al., 2019) in R version 4.02 (R Core Team, 2020).

#### Floral scent

To assess the chemical composition and amount of scent emitted by flowers of *S. longicolle* and *S. acuminatum*, we enclosed inflorescences in polyacetate bags (Kalle, Germany) and used a portable pump (Spectrex, Shakopee, MN, USA) to suck air from the bags at a flow rate of 200 mL/min through a tubular glass filter containing 1.5 mg of tenax and 1.5 mg carbotrap (Merck, Johannesburg, South Africa). At the Kareedouw site, we sampled three inflorescences of each species at midday and again in the evening at 19:00, and at the Krakeel site, we sampled four inflorescences at midday. Samples were thermally desorbed and analyzed using a Bruker gas chromatograph‐mass spectrometer (GC‐MS) (Bruker, Johannesburg, South Africa) with a Carbowax column, following the temperature program and methods described by Johnson et al. (2020). We excluded compounds that were present in similar amounts in control ambient samples. Emission rate of volatiles emitted was calculated from injection of a known amount of methyl benzoate. We compared the emission rates and number of compounds in scent samples from the Kareedouw site using gamma and negative binomial generalized linear models, respectively. We used log link functions for both models and assessed model significance using likelihood ratios. Species, time of day, and their interaction were treated as fixed factors.

### Pollen fates

#### Day vs. night removal of pollinaria

To determine the timing of pollination in the study species, we labeled 14 plants of *S. longicolle* and seven of *S. acuminatum* at the Kareedouw site in 2017 and noted the initial number of pollinaria still present in flowers at 09:00. We then re‐examined the flowers at 18:00 to determine how many of the available pollinaria had been removed during the day. This procedure was repeated at 06:00 the following morning to establish the rate of pollinarium removal during the night and again at 17:00 to obtain a second estimate of the rate of pollinarium removal during the day. We used a binomial (events/trials) generalized linear mixed effects model (GLMM) with a repeated measures design to analyze the effects of species, time period and their interaction on the proportion of available pollinaria removed from flowers. Plant nested within species was treated as a random effect.

#### Pollination success

To establish the overall levels of pollination success in *S. longicolle*, we examined recently wilted flowers on plants from each study population and recorded the number of pollinaria removed from flowers and the presence of pollen massulae on stigmas. We also counted the pollen massulae on stigmas and the number of massulae in pollinaria, which enabled us to calculate the overall pollen transfer efficiency (PTE) in each population. The PTE is the percentage of pollen removed from flowers that is deposited on stigmas (Johnson et al., 2005; Johnson and Harder, 2023). This measure was also obtained for *S. acuminatum* at the Kareedouw site.

#### Pollinator‐mediated self‐pollination

We tracked the fate of stained pollen to obtain an estimate of the fraction of the pollen removed by pollinators that was involved in pollinator‐mediated self‐pollination and to assess whether stained pollen is transferred among species. Mechanical self‐pollination is very rare in *Satyrium* (Van der Niet, 2018) and can easily be visually assessed when self pollinaria are deposited onto the stigma while the viscidia remain in place, something that was never observed in any of the study species. Using the methods developed by Peakall (1989) and modified by Johnson et al. (2005), we injected histochemical stains into the anthers of 26 plants of *S. longicolle* and 21 plants of *S. acuminatum* to stain their pollen. We used rhodamine pink (0.2%) and fast green (1%) for anthers of *S. longicolle* and methylene blue (1%) and gentian violet (premixed medicinal preparation “Alpha”) for anthers of *S. acuminatum*. We stained 21 pairs of closely adjacent *S. longicolle* and *S. acuminatum* plants and an extra five *S. longicolle* plants. Each plant was stained with one color and was at least 10 m from another plant of the same species stained with the same color. After 24 h, we recorded the number of stained pollinaria removed from flowers and examined all stigmas on stained plants and counted the number of stained pollen massulae. Pollen on stigmas that was stained the same color as the plant being examined was assumed to have arisen from pollinator‐mediated self‐pollination. This fraction of removed pollen involved in self‐pollination divided by the overall fraction of pollen that reaches stigmas (PTE) provides a measure of the overall rate of self‐pollination in the population (Johnson et al., 2005). We did not attempt to track export of all stained pollen to other plants in the population as it was not feasible to examine hundreds of stigmas of each species.

### Controlled pollination experiments

We conducted controlled pollination experiments in the Kareedouw and Mountain Drive populations of *S. longicolle* to determine the level of pollinator dependence and effect of pollen from different sources on fruit and seed production. Developing inflorescences (*N* = 9 at Kareedouw and *N* = 12 at Mountain Drive) were enclosed in netting fabric and after flowers reached anthesis they were assigned to the following treatments: unmanipulated to test for autogamy, manual self‐pollination to test for self‐compatibility, manual intraspecific cross‐pollination (using pollen donors at least 5 m from the recipient) as a positive control. In the Kareedouw population, we also included two additional treatments: manual pollination with pollen from *S. acuminatum* and manual pollination with pollen from *S. membranaceum*. In the Mountain Drive population, we included an additional natural pollination treatment of 20 flowers on three unbagged plants. In total, treatments on *S. longicolle* were assigned to 46 flowers on nine plants at Kareedouw and 174 flowers on 15 plants at Mountain Drive. To assess pollination success, we collected mature fruits before full dehiscence, then examined seeds with a light microscope and backlighting at 100× magnification. From each fruit, up to 300 seeds were counted, and reproductive success was quantified as the proportion of successful seeds of the total recorded; successful seeds were readily apparent due to the distinct well‐developed embryo.

We used binomial GLMMs to compare the effects of the treatments on the proportion of flowers that set fruit and the proportion of ovules within fruits that had embryos. We conducted separate GLMMs for the Kareedouw and Mountain Drive experiments due to the unbalanced design (each site had unique treatment groups). Because multiple treatments were conducted within inflorescences, plant individual was treated as a random effect. Because binomial GLMMS with an events/trial structure can be prone to overdispersion, potentially leading to type 1 errors, we added an observation‐level random effect (nested in plant), following the method of Bolker (2015). For plotting, we obtained marginal means with standard errors back‐transformed from the logit scale, and post hoc comparisons of treatments were based on the Dunn–Šidák procedure (Kirk, 1995).

## RESULTS

### Pollinator observations

**Figure 3:**
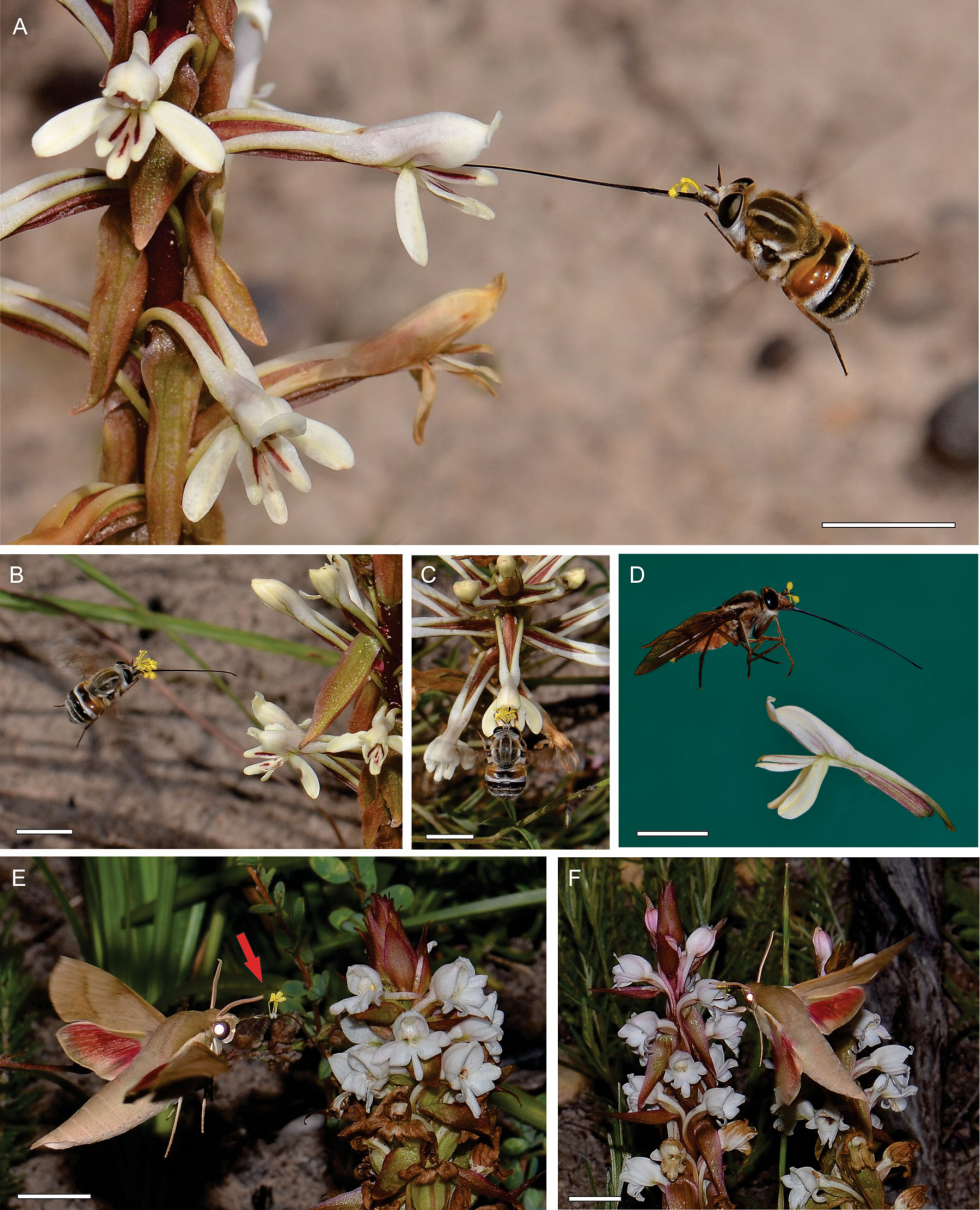
Pollinators of two sympatric *Satyrium* species at the Kareedouw study site. (A) Horsefly *Philoliche gulosa* inserting its proboscis into a floral spur of *S. longicolle*. (B) *P. gulosa* approaching flowers with a large clump of pollinaria of *S. longicolle* on the front of its head. (C) Female *P. gulosa* with proboscis almost fully inserted in one of the twin spurs and pollinaria in contact with the stigma. (D) *P. aethiopica* (Albany form) posed next to a flower of *S. longicolle*. (E) Hawkmoth *Theretra capensis* approaching *S. acuminatum*. Pollinaria (arrowed) have a long caudicle that distinguishes them from pollinaria of *S. longicolle*. (F) *T. capensis* with proboscis inserted into a spur of *S. acuminatum* Scale: 10 mm.

**Figure 4:**
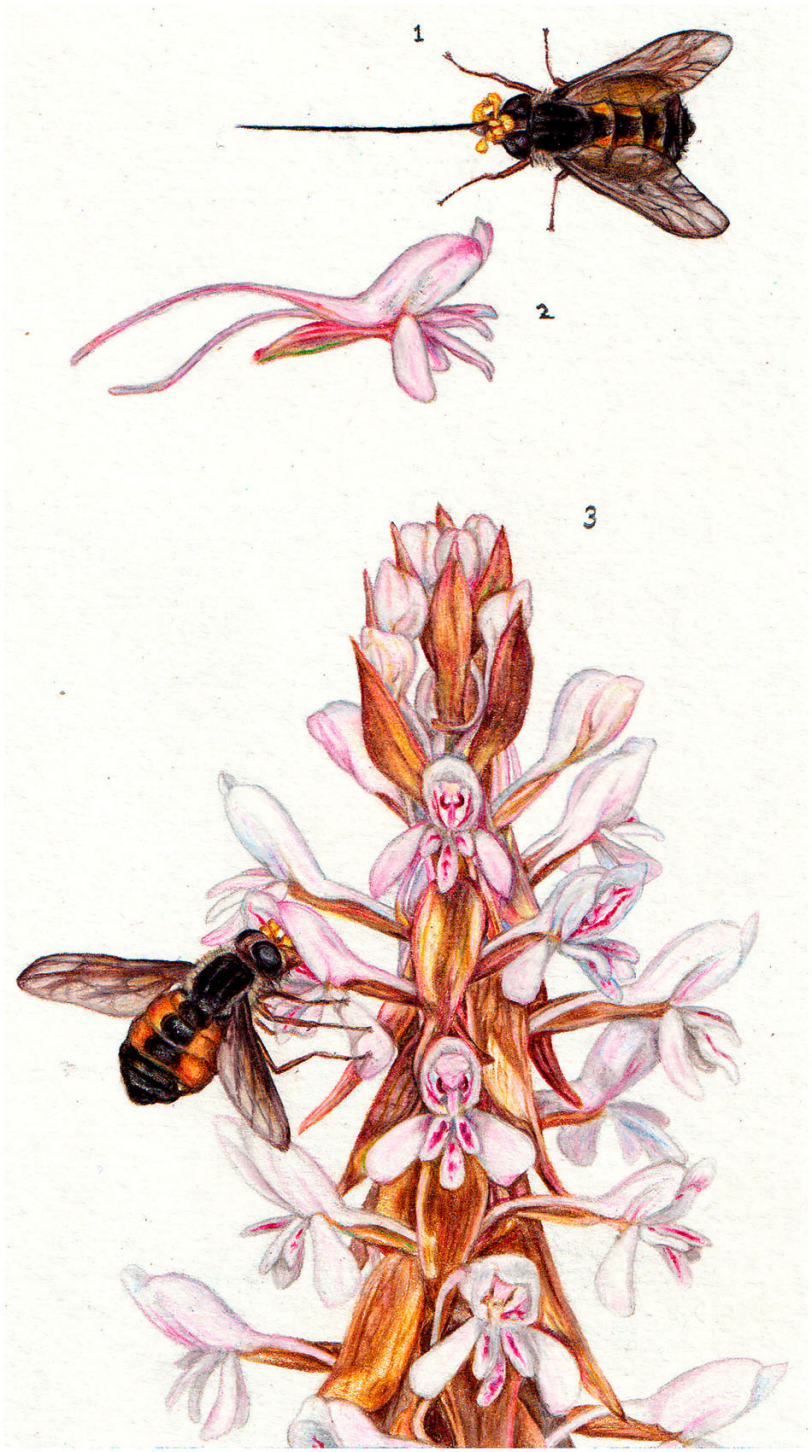
Flowers of *Satyrium longicolle* at the Mountain Drive site visited by the horsefly *Philoliche aethiopica* (Albany form) (3). Upper images show detail of pollinaria of *S. longicolle* attached to the frons of the fly (1) and a flower side view (2). Artwork: Matthew Moir.

Flowers of *S. longicolle* were pollinated almost exclusively by two long‐proboscid horsefly species (Tabanidae), which differed significantly in proboscis length (*χ* ^2^ = 31.7, *P* < 0.001, Table 1). *Philoliche gulosa*, with an effective proboscis length of ca 25 mm, dominated the pollinator assemblages in the Langkloof region (Figure 3), and *Philoliche aethiopica*, with an effective proboscis length of ca 29 mm, dominated the assemblages in the Makhanda region (Figure 4, Table 1). Apart from these flies, the only other visitors recorded were two unidentified female sunbirds and a single *Amegilla* bee that were seen to probe flowers of *S. longicolle* at the Joubertina site. We recorded 165 individual horseflies as visitors to flowers of *S. longicolle*. Of these, 115 flies (69% of the total) were confirmed to carry pollinaria of *S. longicolle* (Figures 3 and 4), 22 (13%) did not carry pollinaria, and the remainder could not be approached closely enough to confirm whether or not they carried pollinaria. Most of the individuals (139) were recorded while visiting *S. longicolle*. In a few cases (16 individuals), flies with pollinaria were recorded while hovering in the vicinity of the orchid. These included males showing territorial behavior and females attracted to our vehicle. Flowers were visited by flies of both sexes, with females making up 57% of the 49 individuals that could be sexed. The sex ratio of fly visitors did not differ from 50:50 (binomial test, *P* = 0.20). Pollinaria were placed dorsally on the frons (forehead) of the flies (Figures 3 and 4), and the median number of pollinaria per fly in the case of individuals (*N* = 53) with pollinaria that could be counted was 3.0 (range = 0–14). Contact between the pollinaria on the flies and the stigma was clearly evident, as seen in video footage (Appendix S2). Pollinaria on captured insects could be reliably identified as those of *S. longicolle* on the basis of the distinct dimensions of the pollinaria (see Floral traits below).

**Table 1:** Horsefly species recorded as visitors to flowers of *Satyrium longicolle*.

				No. individuals recorded per site (confirmed to carry pollinaria)				
Fly species	Body length (mm)	Resting proboscis length (mm)	Extended proboscis length (mm)	KRAK	JOUB	KAREE	MOUN	QUAR
*Philoliche gulosa*	14.4 ± 0.18	17.7 ± 0.27	24.7 ± 0.27	1 (1)	12 (11)	103 (78)	—	—
*Philoliche aethiopica*	19.3 ± 0.37	22.1 ± 0.53	29.4 ± 0.53	—	2 (2)	2 (1)	5 (5)	2 (1)

Size measurements are means (±SE). Site abbreviations: KRAK = Krakeel, JOUB = Joubertina, KAREE = Kareedouw, MOUN = Mountain Drive, QUAR = Quarry.

During their foraging bouts, flies visited multiple inflorescences of *S. longicolle* in the study populations and always ignored plants of *S. acuminatum* and *S. membranaceum*, which were interspersed with those of *S. longicolle*. Given the ca 600:200:80 relative abundances of flowering plants of *S. longicolle*, *S. acuminatum*, and *S. membranaceum* at the Kareedouw site, the fidelity of flies to *S. longicolle* at this site was statistically highly significant (*χ* ^2^ = 64.3, *P* < 0.0001). The mean number of plants visited in each bout (*N* = 46) was 2.23 ± 0.38 (range: 1–13), and the mean number of flowers probed per plant was 2.13 ± 0.24 (range: 1–9). In cases where we were able to ascertain the number of flowers per plant probed by the flies (*N* = 24), we determined that flies probed 33 ± 0.04% of the open flowers available on each plant. The mean duration of flower probing (when the proboscis is inserted into the spur) was 2.23 ± 0.26 s (range: 0.56–6.60 s, *N* = 33), and we observed that flies never probed more than one of the two floral spurs on each flower.

Plants of *S. acuminatum* at the Kareedouw site were visited in the evening by hawkmoths. We recorded five foraging bouts by *Hyles livornica*, three foraging bouts by *Theretra capensis* (Figure 3E,F), and one visit by *Agrius convolvuli*. Pollinaria on these moths had viscidia that were wrapped around the proboscis and had distinctive long caudicles (Figure 3E), indicating that they had originated from *S. acuminatum*. Using the available light at dusk, we were able to follow the hawkmoths as they foraged on *S. acuminatum*, and in all cases, they ignored interspersed plants of *S. longicolle*. Given that plants of *S. acuminatum* made up ca 22.7% of the flowering *Satyrium* plants at the site, the fidelity to this species shown by the nine hawkmoth individuals was statistically significant (*χ* ^2^ = 31.5, *P* < 0.001). Plants of *S. membranaceum* were less abundant at the study sites and were at a later stage of flowering, but we did observe one visit to this species by *T. capensis* (which also visited plants of *S. acuminatum*) and one visit by an unidentified noctuid moth.

### Floral traits

#### Morphology, nectar, and flowering phenology

Floral spur length of *S. longicolle* varied across the distribution range; populations in the west had spurs ca 30 mm long, while those in the east had spurs ca 40 mm long (Table 2). The lengths of the spurs of the moth‐pollinated congeners were very similar overall to those of *S. longicolle* (Table 3). The main difference among species was the angle of the spurs with those of *S. longicolle* being relatively straight, while those of the moth‐pollinated congeners were strongly curved downward and vertical in the distal region (Figure 1, Table 3). The standing crop of nectar in *S. longicolle* flowers was ca 0.7 µL per spur (resulting in 1.4 µL per flower), and the nectar sugar concentration in flowers was approximately 30%. Similar values for both volume and sugar concentration were recorded for the moth‐pollinated congeners (Table 3).

**Table 2:** Floral traits and measures of pollination success in five populations of *Satyrium longicolle* (see Table 1 for population abbreviations).

	Southern Cape			Eastern Cape			
Measure	KRAK	JOUB	KAREE	MOUN	QUAR	*χ* ^2^	*P*
Spur length (mm)	28.7 ± 0.80^a^	30.2 ± 0.51^a^	30.2 ± 0.50^a^	39.1 ± 0.68^b^	42.6 ± 0.81^c^	182.0	<0.001
Nectar volume (µL)	1.2 ± 0.16^a^	0.61 ± 0.14^b^	0.43 ± 0.09^b^	2.0 ± 0.13^c^	1.5 ± 0.13^a^	82.0	<0.001
Nectar concentration (%)	36.3 ± 1.5^a^	33.3 ± 1.6^a^	39.5 ± 0.71^b^	32.9 ± 1.2^a^	27.5 ± 1.2^c^	71.4	<0.001
No. massulae per pollinium	241 ± 38	127 ± 9.8	167 ± 4.4	225 ± 27	―	2.52	0.47
No. massulae on stigmas	44.2 ± 13.2^a^	4.3 ± 0.88^b^	10.7 ± 1.7^c^	13.8 ± 3.9^c^	―	198.3	<0.001
Pollinated flowers (%)	90.4 ± 4.1^a^	24.6 ± 4.0^b^	76.8 ± 5.5^a^	20.5 ± 2.3^b^	24.7 ± 3.4^b^	127.2	<0.001
Pollinaria removed (%)	56.7 ± 6.8	39.4 ± 4.6	48.2 ± 5.6	46.2 ± 3.5	50.0 ± 4.7	5.12	0.27
Pollen transfer efficiency (%)	16.2	5.7	8.8	8.2			

Values are means ± SE. Means that share letters do not differ significantly.

**Table 3:** Floral traits and measures of pollination success in *Satyrium longicolle*, *S. acuminatum*, and *S. membranaceum* in the Langkloof Valley.

Measure	*S. longicolle*	*S. acuminatum*	*S. membranaceum*	*χ* ^2^	*P*
Spur length (mm)	29.7 ± 0.27^a^	25.8 ± 0.50^b^	29.8 ± 1.00^a^	41.5	<0.001
Spur curvature	0.09 ± 0.02^a^	0.26 ± 0.02^b^	0.37 ± 0.03^c^	33.1	<0.001
Spur angle at distal end (°)	30.6 ± 3.3^a^	83.4 ± 2.8^b^	75.3 ± 5.4^b^	45.6	<0.001
Nectar per spur (µL)	0.73 ± 0.05^a^	0.71 ± 0.13^a^	0.39 ± 0.13^b^	6.43	0.04
Nectar concentration (%)	36.3 ± 0.64^a^	30.0 ± 1.49^b^	26.0 ± 1.73^b^	32.9	<0.001
Pollinarium length	2.6 ± 0.09^a^	4.5 ± 0.09^b^	3.6 ± 0.14^c^	53.6	<0.001
Caudicle length	1.7 ± .07^a^	2.7 ± 0.07^b^	2.0 ± 0.11^c^	38.6	<0.001
No. massulae per pollinium	172 ± 36.6^a^	416 ± 131.7^b^	―	5.68	0.017
No. massulae on stigmas	13.8 ± 0.96^a^	7.5 ± 0.85^b^	―	19.2	<0.001
Pollinated flowers (%)	68.4 ± 4.2^a^	55.1 ± 5.3^b^	―	4.02	0.04
Pollinaria removed (%)	48.1 ± 2.3	55.1 ± 3.7	―	2.46	0.11
Pollen transfer efficiency (%)	8.3	1.64	―		

Values are marginal means ± SE with site nested within species. Means that share letters do not differ significantly.

Flowering phenology of *S. longicolle* and *S. acuminatum* almost completely overlapped, while flowering began earlier for *S. membranaceum*, but still overlapped considerably with the other two congeners (Appendix S1).

#### Spectral reflectance

**Figure 5:**
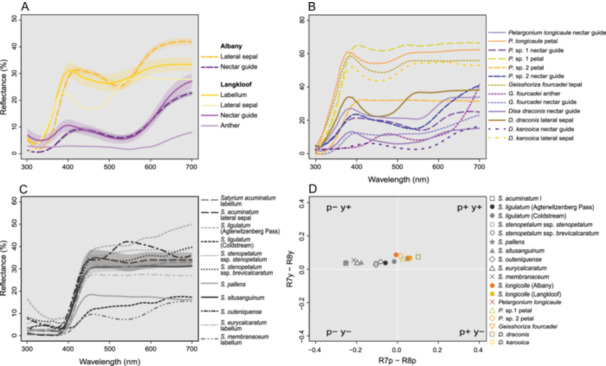
Plots showing spectral reflectance curves measured from floral parts for (A) Albany and Langkloof populations of *Satyrium longicolle*, (B) six species also representing the tabanid horsefly pollination guild, and (C) *Satyrium* species pollinated by nocturnal hawkmoths. The 95% confidence intervals, represented by shaded regions surrounding aggregated reflectance curves, are given for *S. longicolle* and *S. acuminatum*. (D) Spectral loci modeled in Troje color‐space based on the photoreceptor sensitivities of *Eristalis tenax* (Diptera: Syrphidae). Each quadrant represents relative excitation of R7p and R8p, as well as R7y and R8y fly photoreceptor subsystems.

Flowers of *S. longicolle* are light cream to pink in human perception and reflect UV wavelengths (Figure 5A). The nectar guides on the perianth and the anther are dark purple (Figure 5A). Very similar spectra were recorded for flowers of other plants that are also visited by horseflies (Figure 5B). The flowers of the moth‐pollinated congeners *S. acuminatum* and *S. longicolle* are white and pink, respectively, in human perception and are strongly UV‐absorbing. This spectral signature was shared by flowers of other *Satyrium* species that are pollinated by moths (Figure 5C). In a fly vision model, there was a clear pattern of clustering of spectra of fly‐pollinated species that was distinct from that of moth‐pollinated species (Figure 5D). Among the spectral loci of fly‐pollinated species, 71% of pairwise chromatic contrasts were above the functional discrimination threshold of 0.021 Troje units; 100% of the contrasts between fly‐ and moth‐pollinated species exceeded this threshold. Achromatic contrast values for hawkmoth‐ and fly‐pollinated species (Appendix S3) did not differ significantly when modelled in a fly vision system (*F*_1,15_ = 0.61, *P* = 0.45) or in a hawkmoth vision system (*F*_1,15_ = 3.19, *P* = 0.094).

#### Floral scent

To the human nose, flowers of *S. longicolle* are very weakly‐scented, which was confirmed by the GC‐MS analysis that showed that their scent was about 95 times weaker than that of flowers of *S. acuminatum* in the afternoon and about 4800 times weaker in the evening (Table 4). Emission rates differed significantly according to species (*χ* ^2^ = 77.7, *P* < 0.001), time of day (*χ* ^2^ = 22.3, *P* < 0.001), and the interaction between species and time of day (*χ* ^2^ = 19.2, *P* < 0.001). The number of compounds in the floral scent of *S. acuminatum* was 4‐fold greater than in the floral scent of *S. longicolle* (Table 4; Appendix S4). The number of scent compounds differed significantly among species (*χ* ^2^ = 4.89, *P* = 0.03), but did not differ with time of day (*χ* ^2^ = 0.20, *P* = 0.65) or the interaction between species and time of day (*χ* ^2^ = 0.06, *P* = 0.79). The dominant compound in the scent of *S. longicolle*, albeit present in very small amounts, was the benzenoid coumarin, while in *S. acuminatum* the dominant compound was the monoterpene alcohol linalool (Appendix S4).

**Table 4:** Emission rates and number of compounds in the floral scent of *S. longicolle* and *S. acuminatum*. See the section on floral scent in the Results for statistical analysis.

	*S. longicolle*			*S. acuminatum*	
	Kareedouw	Kareedouw	Krakeel	Kareedouw	Kareedouw
Measure	Afternoon	Evening	Midday	Afternoon	Evening
Emission rate (µg/flower/h)	0.04 ± 0.02	0.0005 ± 0.0002	0.07 ± 0.03	3.8 ± 1.80	2.4 ± 1.38
No. compounds	6.3 ± 1.45	4.0 ± 1.15	5.8 ± 1.20	22.7 ± 2.74	20.0 ± 3.16

### Pollen fates

#### Day vs. night removal of pollen

**Figure 6:**
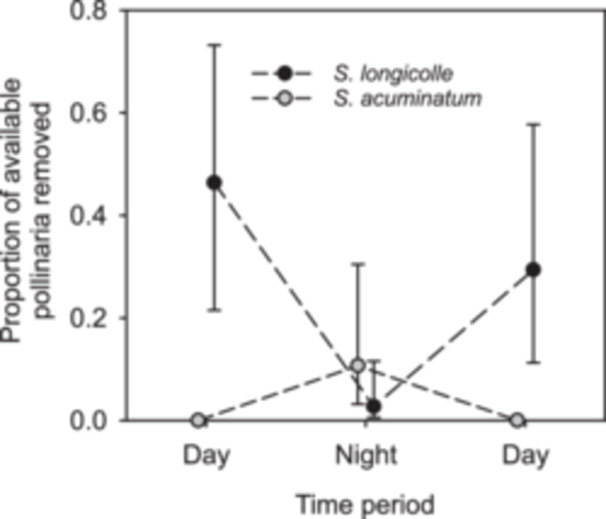
Removal of pollinaria from flowers of *S. longicolle* and *S. acuminatum* during days versus nights.

We recorded patterns of pollinarium removal that were consistent with the observed timing of pollinator activity. Pollinaria of *S. longicolle* were removed only during the day; those of *S. acuminatum* were removed only during the night (Figure 6). Overall levels of pollinarium removal did not differ according to species (*F*_1,19_ = 2.58, *P* = 0.124) or time period (*F*_2,33_ = 1.14, *P* = 0.30), but there was a highly significant species × time period interaction (*F*_2,33_ = 5.65, *P* = 0.008).

#### Pollination success

In the case of *S. longicolle*, we recorded pollen on the stigmas of 21–90% of flowers in the study populations, and the mean number of pollen massulae deposited ranged from 4 to 44 (Table 2). The percentage of pollinaria removed ranged from 39% to 57%, and the overall percentage of pollen removed from flowers that was deposited on stigmas ranged from 5.7% to 16.2% (Table 2). For the moth‐pollinated congener *S. acuminatum*, the proportion of pollinaria removed from flowers and the proportion of pollinated flowers were similar to those of *S. longicolle*, but the overall percentage of removed pollen that was deposited on stigmas (PTE) at the Kareedouw site was 4‐fold less than on *S. longicolle* (Table 3).

#### Pollinator‐mediated self‐pollination

Fifteen stained pollinaria were removed from flowers on seven plants of *S. longicolle*, and 21 stained pollinaria were removed from flowers of 11 plants of *S. acuminatum*. We recorded stained self massulae on eight flowers of *S. longicolle* and nine flowers of *S. acuminatum*. Of the stained pollen massulae removed from flowers, 1.4 ± 1.01% were deposited on self stigmas of *S. longicolle* and 1.5 ± 1.14% on self stigmas of *S. acuminatum*. We did not record any instances of transfer of stained pollen among the species.

### Controlled pollination experiments

**Figure 7:**
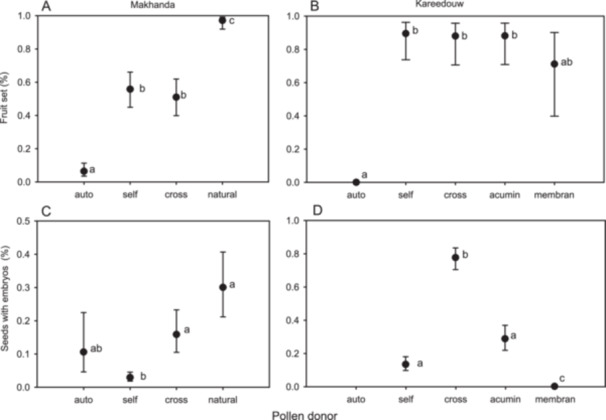
Effects of controlled pollinations on fruit (A, B) and seed set (C, D) in flowers in two populations of *S. longicolle*. acum = manually pollinated with pollen of *S. acuminatum*, auto = unmanipulated to test for autogamy, cross = manually cross‐pollinated, membran = manually pollinated with pollen of *S. membranaceum*, natural = unbagged flowers exposed to natural levels of pollination, self = manually self‐pollinated. Values are marginal (model‐adjusted) means ± SE.

Pollination treatments had statistically significant effects on fruit set at both Makhanda (*F*_3,170_ = 11.36, *P* < 0.001) and Kareedouw (*F*_4,41_ = 4.13, *P* = 0.007). Bagged unmanipulated flowers of *S. longicolle* at Kareedouw did not produce any fruits, and at Makhanda, they produced just a few fruits (Figure 7). It was thus evident that *S. longicolle* is reliant on pollinators for seed production. High levels of fruit set were obtained following both manual self and manual cross pollination (Figure 7). The proportion of ovules that contained embryos in fruits also differed significantly among pollination treatments at Makhanda (*F*_3,63_ = 10.53, *P* < 0.001) and Kareedouw (*F*_3,21_ = 10.53, *P* < 0.001). In both populations, the percentage of ovules that developed into seeds was significantly lower for the manual self‐pollination treatment than for the manual cross‐pollination treatment (Figure 7). Natural levels of fruit set at Makhanda exceeded those of hand‐pollinated flowers, and the percentage of seed set in these fruits was similar to that of cross‐pollinated flowers (Figure 7). Application of pollen of *S. acuminatum* and *S. membranaceum* resulted in fruit formation in *S. longicolle* in the Kareedouw population, but seeds with embryos were only found in fruits arising from application of *S. acuminatum* pollen, albeit at a lower frequency than was the case for intraspecific crosses with pollen of *S. longicolle* (Figure 7).

## DISCUSSION

This study provides evidence for complete separation in the pollination niches of some coflowering *Satyrium* species, which may facilitate their coexistence. This is the first recorded case of floral specialization for pollination by tabanid flies in a rewarding orchid species in general, and for *Satyrium* in particular, and one of only a handful of cases known among orchids worldwide. As documented for other plants pollinated by long‐proboscid flies (Goldblatt and Manning, 1999; Johnson and Steiner, 2003), the pollination system of *S. longicolle* is characterized by extreme specialization with only two fly species involved, as well as very close phenological matching of the flowering time of the orchid and the flight periods of the fly pollinators (Appendix S1). Also consistent with many other long‐proboscid fly pollination systems is the geographical variation in flower depth and the very low rates of fly activity in some, but not all, populations.

Although the pollination system of dozens of species of the African and Asian orchid genus *Satyrium* have been studied, especially those in South Africa (e.g., Johnson, 1996, 1997a; Johnson et al., 2011; Van der Niet et al., 2015), specialization for pollination by long‐proboscid tabanid flies had not been recorded previously. The discovery of a novel tabanid fly pollination system in *Satyrium* was not surprising, given the floral trait syndrome of unscented, long‐spurred flowers that reflect light in the UV region and have contrasting nectar guides. These floral trait combinations are found in species from other plant genera and families for which pollination by tabanid flies has been confirmed (Johnson, 2025b). This correspondence between floral syndrome and observed pollinators provides confirmation that floral traits can be used to predict the pollination guild to which a plant species belong, especially for species with highly specialized pollination systems (Johnson and Wester, 2017).

Most orchids are considered to be self‐compatible (Tremblay et al., 2004), yet manual self‐pollination in *Satyrium* species often results in strong reductions in both fruit and seed production relative to cross‐pollinated flowers (Ellis and Johnson, 1999). This reduction in seed production following selfing was also observed in our experiments with *S. longicolle* (Figure 7) and is most likely the consequence of strong early‐acting inbreeding depression that is also known from other orchid species (e.g., Travers et al., 2018), although we cannot rule out some kind of self‐incompatibility system. Although nectar‐rewarding species such as *S. longicolle* are expected to experience higher levels of pollinator‐mediated self‐pollination than nonrewarding species (Jersáková et al., 2006), we noted that horseflies seldom probed all the open flowers on plants and, for some unknown reason, always probed just one of the two spurs in each flower. By dividing the value of 1.5% obtained for removed stained pollen that was deposited on self stigmas in the Kareedouw population by the value of 8.8% obtained for overall pollen transfer efficiency in this population (Table 1), we obtained a rate of 16% for pollinator‐mediated self‐pollination in *S. longicolle*, which is a relatively low value for a rewarding *Satyrium* species (Jersáková and Johnson, 2007; Van der Niet et al., 2011) and implies that the great majority of seeds would arise from outcrossing. However, this measure should be considered a rough approximation because the estimate for PTE was integrated over a few days and is an estimate for the entire population, while the estimate for the fraction of self‐pollination based on pollen staining was obtained for a shorter time and for fewer plants.

By combining natural history observations of pollinators with trait analyses and experiments, this study helps to clarify aspects of the evolutionary radiation of *Satyrium*. The study adds to the evidence that specialization in pollination systems can allow related *Satyrium* species to coexist and co‐flower within communities (Ellis and Johnson, 1999) and that geographical variation in floral traits in *Satyrium* species can reflect adaptations to different pollinator assemblages (Johnson, 1997b).

### Pollinator‐mediated ethological isolation

Our experiments showed that artificial crosses between *S. longicolle* (parent) and *S. acuminatum* (donor) can result in fruits with seeds containing embryos (Figure 7). Although only an approximate measure of interfertility, given that incompatibilities may arise later in development (i.e., postzygotic incompatibility), the percentage of seeds with embryos following interspecific crosses has been linked with the ability of *Satyrium* species to hybridize (Ellis and Johnson, 1999). One of the constraints of this study was that techniques for germinating seeds of *Satyrium* species and inoculating them with fungal symbionts have not yet been developed in South Africa. Although *S. acuminatum* and *S. longicolle* did not form hybrids at the study sites, both species are known to be involved in hybrid formation with species with which they share some flower visitors. *Satyrium acuminatum* forms hybrids with another moth‐pollinated species, *S. ligulatum*, in a few zones of contact between these largely allopatric species. *Satyrium longicolle* is also known to form natural hybrids with the bird‐pollinated species *Satyrium coriifolium* with which it occurs sympatrically at some sites (Johnson, 2018). The agents of hybridization between *S. coriifolium* and *S. longicolle* are almost certainly sunbirds because *S. coriifolium* is specialized for sunbird pollination (Johnson, 1996) and we observed two cases of sunbirds probing flowers of *S. longicolle*. Horseflies are extremely unlikely to probe the orange flowers of *S. corrifolium* and have been shown experimentally to avoid orange flowers (Jersáková et al., 2012). Given the complete absence of observed hybrids among the study species at any of the field sites, there appear to be strong isolating barriers among them, which include ethological isolation between fly‐pollinated *S. longicolle* and the moth‐pollinated species *S. acuminatum* and *S. membranaceum*.

The remarkable fidelity of the flies to flowers of *S. longicolle* (and of moths to *S. acuminatum* and *S. membranaceum*) when foraging, is likely to contribute to reproductive isolation between *S. longicolle* and sympatric congeners that have highly overlapping flowering periods (Appendix S1). The field‐based observations of pollinator foraging fidelity were supported by the results of our pollinarium removal experiments. Specifically, pollinaria were removed only during the day for *S. longicolle* and during the night for *S. acuminatum* (Figure 6), which is also consistent with published data on the almost exclusively nocturnal removal of pollinaria from flowers of *S. acuminatum* and *S. membranaceum* (Van der Niet et al., 2015; Botes et al., 2020). Although not comprehensively assessed, transfer of color‐labeled pollen never occurred between the species. Complete pollinator‐mediated isolation occurs regularly among co‐occurring orchids, and has been documented in *Disa* (Steiner et al., 1994; Newman and Johnson, 2025), *Ophrys* (Xu et al., 2011), and *Gymnadenia* (Sun et al., 2015). This form of reproductive isolation occurs mainly through divergence in pollinator‐attraction traits of flowers linked to the sensory systems of different pollinators, though studies that link reproductive isolation to analyses of floral traits using insect sensory systems are still very rare (Whitehead and Peakall, 2014; Sun et al., 2015). For example, based on pollen staining experiments used to track interspecific pollen transfer between two *Gymnadenia* species that differ in both scent and flower color, Sun et al. (2015) found only intraspecific pollen transfer. The results of our study indicate that absolute pollinator‐mediated isolation likely occurs through differences in the scent profiles, colors and spur orientation of the sympatric orchids, with a strong role for traits in segregating visitors into day versus night periods. Specifically, *S. acuminatum* is characterized by emission of very large amounts of linalool, benzyl alcohol, and eugenol (Appendix S4), which are associated with pollination by moths (Bischoff et al., 2014), whereas *S. longicolle* emits very little scent which is typical for plants pollinated by long‐proboscid flies (Campbell et al., 2016; Johnson et al., 2025). The spectral reflectance of flowers is also very different in these orchid species, with UV‐reflectance in flowers of *S. longicolle* and UV‐absorbance in flowers of *S. acuminatum* and *S. membranaceum*. These spectral differences are likely apparent to both sets of pollinators and may be used for discrimination by both moths and flies. Although we found no differences in achromatic contrast values for fly‐ and moth‐pollinated species in fly and moth vision, respectively (Appendix S3), it is likely that both sets of pollinators use achromatic contrast for flower detection under some circumstances (An et al., 2018; Van Der Kooi and Kelber 2022). The underlying physiological basis and role of achromatic contrast in flower discrimination by insects is still very poorly understood, particularly for flies. What is more certain is that flies use overall color differences (and possibly also morphological differences) to discriminate among flowers in the two plant pollination guilds (Figure 5). There is also evidence that hawkmoths can use color information when foraging in near darkness (Kelber et al., 2002) and would be able to use that ability to discriminate among the species. Although the spurs of all three *Satyrium* species are similar in length and contain similar amounts of nectar, horseflies have a proboscis that is fixed in a forward position and would not be able to efficiently probe the strongly decurved spurs of the two moth‐pollinated *Satyrium* species. We also observed that hawkmoths avoided flowers of *S. longicolle*. Given that the flower spur lengths are very similar among all of these sympatric *Satyrium* species and have similar levels of achromatic contrast, we suspect that moths discriminate using color information and differences in the amount and composition of floral scent. It may also be the case that, given a choice, they prefer to avoid feeding on flowers with horizontal tubes, as manipulation of flowers from a vertical to a horizontal angle has been shown to discourage visits by hawkmoths (Campbell et al., 2016), even though moths have a flexible proboscis and can, if necessary, feed on flowers with horizontal tubes.

Although not the central focus of this study, the lack of hybrids between *S. acuminatum* and *S. membranaceum* is noteworthy because moths were observed to move between plants of these species and their respective pollinaria are placed on the same part of the proboscis of moths. Genetic barriers to hybridization between *S. membranaceum* and the other two species seem likely given that *S. membranaceum* is rather distantly related to them (Van der Niet and Linder, 2008), a factor that may be correlated with genetic compatibility (Moyle et al., 2004). Genetic distance may also explain why our crosses between *S. longicolle* and *S. membranaceum* did not yield seeds (Figure 7).

### Pollination ecotypes?

In South Africa, intraspecific geographical variation in floral tube length of long‐proboscid fly‐pollinated plants is common and associated with either covariation in proboscis length within the same fly species, potentially owing to different local coevolutionary processes (Anderson and Johnson, 2008; Pauw et al., 2009), or with pollinator shifts between different long‐proboscid fly species that differ in proboscis lengths (Johnson and Steiner, 1997; Pauw et al., 2009; Newman and Johnson, 2021). In the latter case, these geographical shifts may be associated with regional differences in the niche requirements of the different pollinator species (Duffy and Johnson, 2017; Kay and Anderson, 2025; Johnson, 2025b). Although the proboscis lengths of the pinned flies that we measured in this study were about 30% shorter than the floral spurs, these flies are known to be able to extend the length of their proboscis by about the same percentage when feeding (Morita, 2011). If we use the functional (i.e., extended) length of the proboscis (Table 1), we find a close correspondence with the spur length of *S. longicolle* (Table 2). This trait matching is expected given that pollinaria are placed on the head of the pollinator, which means that a proboscis that is longer than the spur would fail to result in contact between the head and viscidia, potentially resulting in strong selection on spur length. *Philoliche gulosa*, which has a relatively short proboscis, made up the great majority of the flies we observed in the western *S. longicolle* populations (Table 1, Figure 3A–D), while the Albany form of *P. aethiopica*, which has a longer proboscis, was the only fly we recorded in the eastern part of the distribution of *S. longicolle* (Table 1, Figure 4). Variation in the floral spur length of *S. longicolle* from shorter spurs in the west to longer spurs in the east (Table 2), thus appears to reflect a natural geographical gradient in horsefly assemblages (Figure 2). There is some uncertainty about whether *P. gulosa* occurs in the eastern part of the distribution range of *S. longicolle*. Although there are dozens of museum and iNaturalist records of this fly species from the western part of the distribution of *S. longicolle* (Johnson, 2025a), there is only a single record of fly species from the Makhanda (Grahamstown) region, a museum specimen with a label indicating that it was collected by R. C. Wood on 16 December 1936. Despite the existence of this specimen, Usher (1972) expressed doubts about whether *P. gulosa* occurs in the Makhanda region. Given that there are no other records of this fly from the Makhanda region, we infer that the fly is either extremely rare in this region or that Wood mislabeled a specimen that he had collected when travelling east of Makhanda before 16 December 1936. Overall, divergence in floral traits associated with different pollinator assemblages in different parts of the range of *S. longicolle* suggests the formation of pollination ecotypes in this species (Johnson, 2025b). However, we did not obtain rigorous evidence for pollination ecotype formation, such as experiments that assess the fitness consequences of traits in different environments (Newman et al., 2015). Further study would therefore be required to confirm that spur length variation in *S. longicolle* is a consequence of local adaptation to different long‐proboscid fly species.

## CONCLUSIONS

Despite intensive study of pollination systems in *Satyrium*, new pollination systems continue to be discovered, and the asymptote has not yet been reached. The reasons for the slow progress in uncovering all the pollination systems in the genus are complex, but chief among them is the tendency of many species in the fynbos region to flower only at intervals of 10–20 years after fires. We often had to wait for several years between opportunities to study flowering populations of *S. longicolle* in the Langkloof region. Ultimately, it required more than a decade for us to gather sufficient information to document the pollination system of this particular species. Unfortunately, the available phylogenetic data for *Satyrium* (Van der Niet and Linder, 2008) is not sufficiently well resolved for us to interpret the ancestral pollination system from which the shift to pollination by tabanid flies occurred in *Satyrium*. Given the predominance of moth pollination in the genus, a shift from moth pollination seems the most likely scenario, but the closely related *S. erectum* is pollinated by bees and has globose viscidia which are also placed on the head, meaning that we cannot rule out a shift from a bee pollination system, or even from a pollination system that is not represented in the extant related species. It will require more focused study of the pollination systems of other congeners and genomic data to provide better species‐level resolution of the phylogeny to better understand radiation and species coexistence in this orchid genus.

## AUTHOR CONTRIBUTIONS

S.D.J.: conceptualization, data acquisition, formal analysis, visualization, chemical analyses, photographic documentation, writing original draft, review, and editing. M.M.: data acquisition, formal analysis, visualization, review and editing. E.N.: data acquisition, review and editing. T.V.N.: data acquisition, review and editing.

## Supporting information


**Appendix S1:** Phenology of the study plant species and the horsefly pollinators of *Satyrium longicolle*.

**Appendix S2:** Video of visits to *Satyrium longicolle* by the tabanid fly *Philoliche gulosa*.

**Appendix S3:** Putative achromatic contrast values (flower vs. background foliage) in moth and fly vision systems for flowers pollinated either by tabanid flies or by hawkmoths.

**Appendix S4:** Relative abundance (%) of compounds in the floral scent of *Satyrium acuminatum* and *S. longicolle*.

## Data Availability

Data are available at Zenodo: https://zenodo.org/records/18958482.
