# Supplementary material for: Pollination by long‐proboscid horseflies and its implications for reproductive isolation among coflowering Satyrium orchids in South Africa
Source: Am J Bot. 2026 Jun 12;113(6):e70221. doi: 10.1002/ajb2.70221 (PMC13280966; doi:10.1002/ajb2.70221)

**Appendix S1.** Phenology of the study plant species and the horsefly pollinators of *Satyrium longicolle*. The number of iNaturalist observations and museum records used is in parentheses*.*


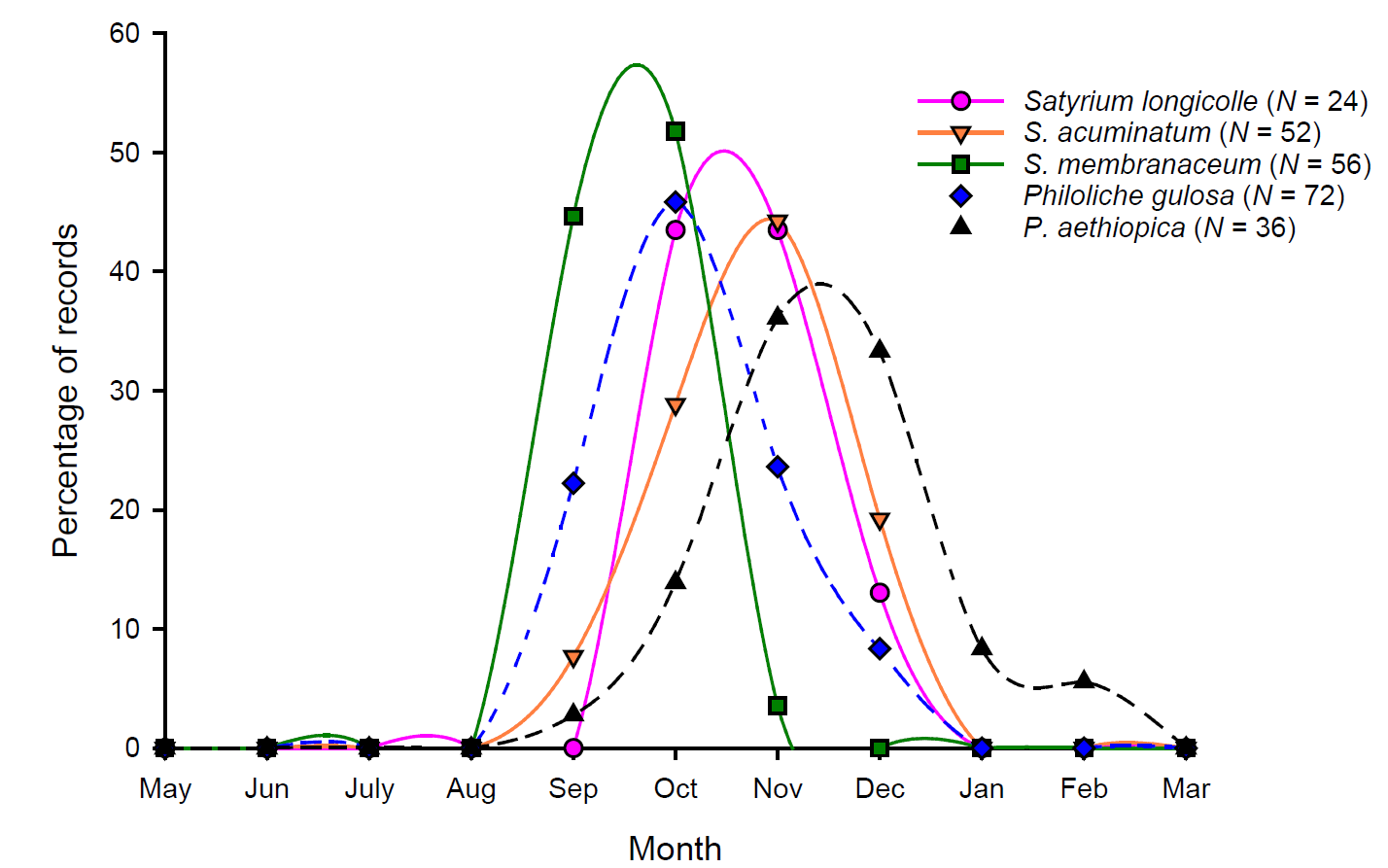

Supplement: Supplementary file 1 — Appendix S1: Phenology of the study plant species and the horsefly pollinators of Satyrium longicolle. [file AJB2-113-e70221-s004.docx]
