# Supplementary material for: Pollination by long‐proboscid horseflies and its implications for reproductive isolation among coflowering Satyrium orchids in South Africa
Source: Am J Bot. 2026 Jun 12;113(6):e70221. doi: 10.1002/ajb2.70221 (PMC13280966; doi:10.1002/ajb2.70221)

**Appendix S3.** Putative achromatic contrast values (flower vs background foliage) in (A) a moth vision system and (B) a fly vision system for flowers pollinated by the horsefly *Philoliche gulosa* (yellow symbols) versus flowers pollinated by hawkmoths (grey symbols). Values are means (±SE). See section Spectral reflectance in Results for statistical analysis.


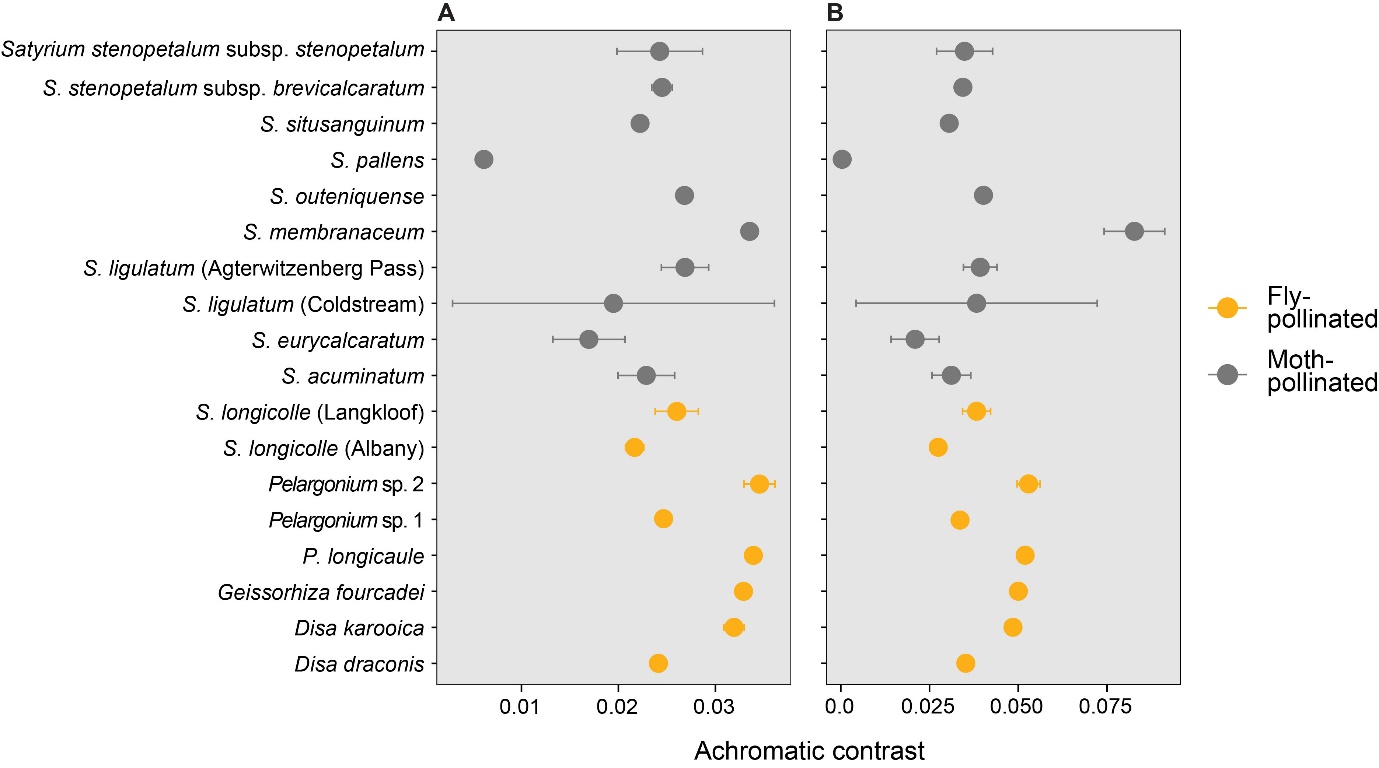

Supplement: Supplementary file 3 — Appendix S3: Putative achromatic contrast values (flower vs. background foliage) in moth and fly vision systems for flowers pollinated either by tabanid flies or by hawkmoths. [file AJB2-113-e70221-s003.docx]
