# Supplementary material for: Pollination by long‐proboscid horseflies and its implications for reproductive isolation among coflowering Satyrium orchids in South Africa
Source: Am J Bot. 2026 Jun 12;113(6):e70221. doi: 10.1002/ajb2.70221 (PMC13280966; doi:10.1002/ajb2.70221)
Supplement: Supplementary file 4 — Appendix S4: Relative abundance (%) of compounds in the floral scent of Satyrium acuminatum and S. longicolle. [file AJB2-113-e70221-s002.docx]

**Appendix S4.** Mean ± SE relative abundance (%) of compounds in the floral scent of *S. acuminatum* and *S. longicolle* measured using gas chromatography coupled with mass spectrometry. RI = retention index. Compounds in bold were the most abundant in the floral scent bouquet of the respective species. Unknown compounds are listed with the most frequent mass fragments (*m*/*z*) in descending order of abundance.

|  |  |  | ***S. longicolle* (%)** | | | ***S. acuminatum* (%)** | |
| --- | --- | --- | --- | --- | --- | --- | --- |
|  |  |  | Kareedouw | Kareedouw | Krakeel | Kareedouw | Kareedouw |
| **RI** | **Compound class** | **Compound** | Afternoon | Evening | Midday | Afternoon | Evening |
| 1167 | Monoterpene | β-myrcene | ― | ― | ― | 0.39 ± 0.05 | 0.91 ± 0.48 |
| 1206 | Monoterpene | Limonene | ― | ― | ― | 0.05 ± 0.02 | 0.23 ± 0.17 |
| 1237 | Monoterpene | (*Z*)-β-ocimene | ― | ― | ― | 0.09 ± 0.01 | 0.2 ± 0.09 |
| 1251 | Monoterpene | (*E*)-β-ocimene | ― | ― | ― | 0.18 ± 0.02 | 0.24 ± 0.17 |
| 1319 | Aliphatic alcohol | Hexan-1-ol | ― | ― | 38.57 ± 7.77 | ― | ― |
| 1349 | Aliphatic alcohol | (*E*)-Hex-3-en-1-ol | ― | ― | 7.03 ± 5.24 | ― | ― |
| 1402 | Aliphatic alcohol | Oct-1-en-3-ol | ― | ― | 4.89 ± 1.98 | ― | ― |
| 1423 | Monoterpene | (*Z*)-Linalool oxide (furanoid) | ― | ― | ― | 0.17 ± 0.01 | 0.08 ± 0.01 |
| 1428 | Aliphatic alcohol | *n*-Heptan-1-ol | ― | ― | 10.29 ± 3.45 | ― | ― |
| 1468 | Monoterpene | (*E*)-Linalool oxide (furanoid) | ― | ― | ― | 0.03 ± 0.02 | 0.06 ± 0.03 |
| 1534 | Benzenoid | Benzaldehyde | ― | ― | ― | 0.08 ± 0.03 | 0.02 ± 0.02 |
| 1547 | Monoterpene | Linalool | 6.85 ± 4.92 | 13.23 ± 3.96 | 9.97 ± 3.67 | **93.6 ± 1.59** | **93.64 ± 1.9** |
| 1606 | Monoterpene | Hotrienol | ― | ― | ― | 0.04 ± 0.01 | 0.07 ± 0.05 |
| 1684 | Aliphatic ester | Lavender lactone | 6.03 ± 7.25 | 3.86 ± 6.68 | ― | 0.02 ± 0.0 | 0.07 ± 0.05 |
| 1695 | Benzenoid | 2-Hydroxybenzaldehyde | 9.15 ± 2.54 | 33.5 ± 17.86 | ― | ― | ― |
| 1701 | Monoterpene | (-)-α-Terpineol | ― | ― | ― | 0.08 ± 0.0 | 0.06 ± 0.0 |
| 1714 | Unknown | *m*/*z*: 121,136,65,93,43,63,39,122,53,50, | 10.73 ± 3.91 | 2.04 ± 3.54 | ― | ― | ― |
| 1744 | Monoterpene | (*E*)-Linalool oxide (pyranoid) | ― | ― | ― | 0.01 ± 0.0 | 0.02 ± 0.0 |
| 1765 | Monoterpene | (*Z*)-Linalool oxide (pyranoid) | ― | ― | ― | 0.06 ± 0.0 | 0 ± 0.0 |
| 1801 | Monoterpene | Nerol | ― | ― | ― | 0.03 ± 0.0 | 0.03 ± 0.01 |
| 1845 | Monoterpene | Geraniol | ― | ― | ― | 0.01 ± 0.0 | 0.04 ± 0.01 |
| 1886 | Benzenoid | Benzyl alcohol | ― | ― | ― | 1.29 ± 0.48 | 0.64 ± 0.07 |
| 1920 | Benzenoid | Phenylethyl alcohol | 2.11 ± 0.61 | 1.14 ± 1.97 | ― | ― | ― |
| 1941 | Monoterpene | 2,6-Dimethylocta-3,7-diene-2,6-diol | ― | ― | ― | 0.14 ± 0.07 | 0.02 ± 0.0 |
| 2166 | Benzenoid | Eugenol | ― | ― | ― | 3.38 ± 1.32 | 3.44 ± 2.66 |
| 2247 | Benzenoid | (*Z*)-Iso-eugenol | ― | ― | ― | 0.05 ± 0.01 | 0.01 ± 0.02 |
| 2274 | Benzenoid | Cinnamyl alcohol | ― | ― | ― | 0.02 ± 0.0 | 0.01 ± 0.01 |
| 2286 | Benzenoid | Hydrocoumarin | 1.47 ± 0.93 | ― | ― | ― | ― |
| 2332 | Benzenoid | (*E*)-Isoeugenol | ― | ― | ― | 0.09 ± 0.03 | 0.14 ± 0.2 |
| 2441 | Benzenoid | Coumarin | **63.7 ± 12.64** | **46.2 ± 22.93** | **29.3 ± 14.38** | ― | ― |
| 2538 | Benzenoid | Vanillin | ― | ― | ― | 0.19 ± 0.05 | 0.06 ± 0.05 |
| 2633 | Benzenoid | Vanillyl methyl ketone | ― | ― | ― | 0.03 ± 0.0 | 0.01 ± 0.01 |
